# Praise Is for Actions That Are Neither Expected nor Required

**DOI:** 10.1177/01461672241289833

**Published:** 2024-10-17

**Authors:** Rajen A. Anderson, Shaun Nichols, David A. Pizarro

**Affiliations:** 1 Leeds University Business School, University of Leeds, Leeds, UK; 2 Department of Philosophy, Cornell University, Ithaca, NY, USA; 3 Department of Psychology, Cornell University, Ithaca, NY, USA

**Keywords:** praise, moral judgment, social signals, norms

## Abstract

In six studies, we examined two foundational questions about moral praise. First, what makes an action praiseworthy? In Study 1, participants reported that actions that exceed duties (compared with dutiful actions) deserve greater praise and are perceived as less likely to happen. Second, what do observers infer from praise? Praise may communicate information about local norms. In Study 2, we found that—in general—participants expect praise to increase the likelihood of a behavior. However, in Studies 3–6, participants inferred that moral behavior that receives praise is less common and is less required and expected of people. These inferences led individuals to judge that someone would be *less* likely to perform a behavior that was praised. These studies provide insight into the lay beliefs and communicative function of moral praise.

One of the most fundamental ways in which humans evaluate and organize their social world is by assessing the moral qualities of those around them (Fiske, 2018; Goodwin et al., 2014; Hartley et al., 2016). To do this, our moral systems employ a variety of cognitive tools, including affectively intense rules about how people should (and should not) behave (Nichols, 2002) and social evaluations of responsibility like blame and praise to regulate behavior (Malle et al., 2014).

In the present studies we examined the role of moral praise. Praise has been relatively understudied in moral psychology (Anderson et al., 2020), despite its frequent occurrence across a variety of different social contexts. Parents praise their children for good behavior, managers praise their employees for exceeding their duties, and the media and the public praise individuals after acts of heroism. We sought to address two foundational questions regarding moral praise. First, what sorts of actions generate praise? That is, beyond being “morally good,” is there some unifying dimension that connects actions that are seen as praiseworthy? Second, what inferences do people make from observing judgments of praise? Do they infer that the behavior being praised was obligatory, or that it is supererogatory (i.e., above and beyond what is required)? We hypothesized that praise would provide information on the norms of a situation—that is, beliefs about how statistically common a behavior is and how expected it is of people (Bicchieri & Xiao, 2009).

## Moral Praise, Obligation, and Supererogatory Behavior

Praise has been characterized by some philosophers as indicating that an action is “laudable” (Smith, 1991), and that an agent is praiseworthy when she performs a morally good action for a morally worthy motive (Arpaly & Schroeder, 1999). Building on this work in philosophy, we define moral praise as “a cognitive appraisal regarding an agent’s positive moral behavior and character” (Anderson et al., 2020, p. 694). Praise is distinct from simple liking in that it is about the morality of the target (as opposed to the general positivity of the target). One important function of moral feedback—including praise and blame—is to regulate and promote moral behavior in the future (Curry et al., 2019; Gray et al., 2012; Haidt, 2007). Praising not just positive moral actions, but positive moral actions that were performed with good intentions, may serve the function of encouraging positive actions in the future. But not all positive moral actions may be viewed as deserving of praise—it may be the case that individuals only view actions that are above and beyond the call of duty as praiseworthy.

Philosophers (and social scientists in turn) have distinguished between different conceptualizations of morality, focusing on norms for acts that are required or forbidden of people (i.e., obligatory) and for acts that are simply encouraged or discouraged of people (i.e., supererogatory; Wiltermuth et al., 2010). This distinction is consistent with Immanuel Kant’s conceptualization (1785/1993) of perfect duties (those that are blameworthy if not fulfilled, e.g., caring for one’s child) and imperfect duties (those that are praiseworthy if fulfilled but are not obligatory, e.g., donating a kidney to a stranger). Consistent with this framing, research has highlighted how adults frequently think of helping behavior as good but not obligatory (Dahl et al., 2020).

A related concept in philosophy and theology—originating in the Roman Catholic Church—is that of supererogation (Flescher, 1994; Urmson, 1958). Supererogation refers to conduct that is morally good but not strictly required by one’s typical duties, responsibilities, and obligations. What actions count as supererogatory depend on the norms of a situation and the roles of the moral agent. For example, consider the act of saving another person’s life. By itself this action would likely be considered morally good. However, the extent to which that action is considered supererogatory likely depends on *who* performed the action. If this act were performed by a physician, given the norms assigned to that profession, it would be unlikely to qualify as supererogatory (morally beneficial though the action may be). However, if the act were performed by an engineer who lacks similar norms of duty because of their profession, saving a life would be more likely seen as supererogatory. This framing echoes research showing that people evaluate positive behaviors depending on the perceived statistical norms surrounding those behaviors (Ngo et al., 2015). This suggests that judgments for positive actions (e.g., praise) may depend on how common those actions appear to be, and actions that exceed an agent’s typical duties and responsibilities are likely seen as less common and more praiseworthy.

## Moral Judgments as Social Signals

Moral judgments such as praise and condemnation appear to communicate not only information about the person making the judgment, but also information about the underlying norms guiding such judgments. Observers treat condemnation of immorality as signaling that the condemner possesses good moral character and would behave morally (Jordan et al., 2016, 2017). Emotional reactions to moral events (e.g., guilt for accidents) can provide important information about the agent’s character and future behavior (Anderson et al., 2021; Frank, 1988; Prinz, 2004). In achievement contexts (e.g., academic performance), praise has been shown to signal expectations and norms regarding minimally acceptable behaviors (Chestnut & Markman, 2018; Zhao et al., 2017, 2020). In the moral domain, while past research has found that praise can communicate expectations for future behavior in children (Bryan et al., 2014; Foster-Hanson et al., 2020), to our knowledge there has been no direct work examining inferences of broader moral norms from praise. Given that praise communicates norms in the achievement domain, we suspected that it would in the moral domain as well.

We sought to investigate whether praise would provide normative information, particularly when there is an absence of knowledge about the moral norms. We predicted that if praise is typically provided in response to supererogatory behaviors, observers may infer that a behavior that is praised is not required. Given that behavior in general is influenced by perceived norms (Gelfand et al., 2017), praise may therefore have a counterintuitive effect—that of reducing the frequency or magnitude of the praised behavior in the future because it communicates that the action is not required by the prevailing norm. Such an effect would be particularly surprising, given that people frequently associate praise with the reinforcement of moral behavior (Robbins et al., 2021). However, in situations where there may be reasons to not behave prosocially (e.g., keeping money for oneself vs. giving to another), individuals may feel licensed to behave less prosocially following praise.

## Overview

In Study 1, we examined the role of duties and actions that exceed duties (i.e., supererogatory actions) in the application of moral praise. Consistent with recent theorizing (Anderson et al., 2018, 2020), we hypothesized that people would be more likely to judge a supererogatory action as praiseworthy compared with an action that merely fulfills one’s duty. In Studies 2–6, we investigated the inferences people make about an action when they see an agent receiving praise. In Study 2, we examined general lay beliefs regarding whether people think praise will increase the likelihood of a behavior. In Studies 3–6, we tested the hypothesis that if supererogatory actions are judged as more praiseworthy than obligatory (i.e., duty-bound) actions, participants would infer supererogation and obligation based on the presence or absence of praise for an action. Praise may make an action seem less common, required, and expected of people (Studies 3–6), which may then impact future moral judgment and behavior (Studies 4–6). All studies received IRB approval. We report all manipulations, measures, and exclusions in these studies. Unless otherwise noted, sample sizes were determined before data collection using G*Power (Faul et al., 2009). All materials, data, analysis codes, and preregistrations can be found at https://osf.io/bf5cw. We note all preregistration deviations in the Supplementary Materials.

## Study 1

In Study 1, we investigated people’s lay intuitions regarding the connection between praise and norms and serves as an empirical test of whether praise is more appropriate for supererogatory acts (Anderson et al., 2018, 2020). Participants first listed two behaviors, one they considered as falling within a person’s duties and one they considered as exceeding a person’s duties. We hypothesized that people would be more likely to rate behaviors that exceed duties (i.e., were supererogatory) as more praiseworthy than behaviors that are duties.

We also conducted two exploratory sets of analyses to contextualize how people think about moral praise. First, we examined the associations between people’s judgments regarding their personal likelihood of performing the action, whether people should do the action, praise for doing the action, and blame for not doing the action. Compared with the blameworthiness for failing to act, one possibility is that the praiseworthiness for acting is less connected to people’s own assessments of their personal likelihood for doing the action and their judgments of whether people should do the action. Second, we coded what participants listed as actions to examine whether the types of actions listed as duties versus exceeding duties differed in meaningful ways. One possible difference was in whether such actions involved social targets. Given that morality is theorized to regulate social interactions (Curry et al., 2019; Greene, 2015; Haidt, 2007; Rai & Fiske, 2011; Tomasello & Vaish, 2013), we predicted that participants would be more likely to mention social targets for actions that exceed duties (i.e., more praiseworthy actions) compared with actions that are duties (i.e., less praiseworthy actions). In addition, we further examined whether social targets (if listed) would be more socially distant (e.g., a stranger) for actions that exceed duties than actions that are duties (McManus et al., 2020).

### Methods

#### Participants

We recruited 150 U.S. participants (*M*_age_ = 36.30; 47 women, 101 men, two did not disclose) from Amazon’s Mechanical Turk using CloudResearch’s prime panels (Chandler et al., 2019) and provided them with monetary compensation. We did not have clear expectations of our anticipated effect size; we set our sample size a priori to detect a small-medium effect size (within-subjects comparison of *d* = .3) with power = .95. Most participants (~83%) self-identified their ethnicity as “White.” There were no exclusion criteria.

#### Procedure

After giving consent, participants were presented with introductory text about the study, describing how “some acts are seen as a duty” while “other acts are seen as going beyond duty and obligation” (for full text, see OSF link). We then asked participants to list an action that they considered a duty and an action that they considered above and beyond duty. After participants listed the two actions, we asked participants to make four ratings (presented in random order) about each action (each action was on a separate page, presented in random order). Participants made judgments about the likelihood that they would perform the action if they were in the appropriate situation (*1 Not at all likely* to *7 Extremely likely*) and whether people should perform the action if they were in the appropriate situation (*1 Not at all* to *7 Very much*). Participants also reported how much praise someone would deserve if they were in the appropriate situation and performed the action and how much blame someone would deserve if they were in the appropriate situation and did not perform the action (both from *1 None at all* to *7 A great deal*).

To investigate potential differences in what actions people listed as duties and as above and beyond duty, three research assistants coded the actions that participants listed based on two criteria. First, they coded whether participants explicitly mentioned a social target as the beneficiary of the action (coded as 1, e.g., “helping a stranger”) or not (coded as 0, e.g., “picking up litter on the ground”; ICC_duty_ = .85, ICC_above_ = .40). The coders were instructed to code self-directed actions (e.g., “taking care of yourself) as non-social. Second, if participants listed a social target, they coded those targets based on their relative social proximity to the agent (ICC_duty_ = .53, ICC_above_ = .90): extremely close (coded as 3, e.g., immediate family and similar close others; “helping your parents”), somewhat close (coded as 2, e.g., friends, acquaintances, and neighbors; “helping my neighbor bring in groceries”), or distant (coded as 1, e.g., strangers and generic others; “giving to the needy”). Disagreements between the three coders were resolved by a fourth research assistant, who received the same set of instructions and the ratings from the other three coders.

### Results

#### Primary Analyses

We first examined whether there were differences between participants’ ratings for duties and actions that exceed duty (see Table 1). Participants consistently judged the two types of actions differently. Relative to duties, participants said actions that exceeded duties were behaviors they were less likely to perform, that people have less obligation to perform them (i.e., lower *should* ratings), deserve greater praise for doing so, and deserve less blame for failing to do so, *p*s < .001.

**Table 1.** Study 1 Results.

	Duties	Above and Beyond	
Likely to do the action	6.52 (0.88)	5.11 (1.73)	*t*(148) = 8.92, *p* < .001, *d* = .73
People should do the action	6.35 (1.11)	5.26 (1.57)	*t*(144) = 8.19, *p* < .001, *d* = .68
Praise for doing	3.84 (1.96)	5.53 (1.58)	*t*(148) = 9.25, *p* < .001, *d* = .76
Blame for not doing	4.81 (2.01)	3.26 (1.93)	*t*(148) = 7.40, *p* < .001, *d* = .60

*Note*. Means, SDs, and paired sample *t*-tests comparing ratings for actions that are duties and actions that are above and beyond duty.

#### Correlations

To assess whether judgments of praise or blame were more closely linked to judgments of likelihood and whether people should do an action, we next examined the correlations between people’s judgments regarding duties compared with actions that exceed duty (see Table 2). When evaluating both types of actions, participants’ ratings of their own likelihood of performing the action positively correlated with their ratings of how much people should do the action, *p*s < .001. Praise judgments did not consistently correlate with the other judgments for either type of action—praise was only significantly correlated with blame for not doing the action when the action was a duty, *p* = .003. On the other hand, blame for not doing the action correlated with likelihood judgments and should judgments for both types of actions, *p*s < .03.

**Table 2.** Study 1 Correlations.

	Actions that are duties				Actions that are above and beyond duties			
	Should	Praise	Blame	Praise vs. Blame	Should	Praise	Blame	Praise vs. Blame
Likelihood	.56***	-.005	.18*	*Z* = 1.81, *p* = .07	.59***	.12	.48***	*Z* = 3.51, *p* < .001
Should		-.08	.22**	*Z* = 2.87, *p* = .004		.15	.40***	*Z* = 2.37, *p* = .02
Praise			.24**				.13	

*Note*. Correlations between different judgments and Fisher *Z* test for whether judgments of likelihood and should were more closely correlated with praise or blame judgments.

* *p* < .05, ** *p* < .01, ** *p* < .001.

As exploratory follow-up analyses, we tested whether participants’ judgments of likelihood of doing the action and how much people should do the action were more closely correlated with praise for doing or blame for not doing each type of action (see Table 2). Compared with judgments of praise, participants’ ratings of likelihood were more correlated with blame for not doing the action, nonsignificant but marginally for duties, *p* = .07, and significantly for above and beyond duties, *p* < .001. Similarly, for both duties and above and beyond duties, participants’ judgments of whether people should do the action were more closely correlated with how much blame people deserve for not doing the action than how much praise people deserve for doing the action, *p*s < .02.

#### Text Analysis

We next examined potential differences in what sorts of actions participants listed as duties versus actions that are above and beyond duties. As predicted, we found that participants were more likely to list social targets for actions that are above and beyond duties (78%) than for duties (45%), *p* < .001. When examining only those cases where participants listed a social target for duties, we found that those actions tended to be directed at extremely close targets (87%) relative to somewhat close (4%) or distant (9%) targets. However, when there was a social target for actions that were listed as above and beyond duties, those actions tended to be directed at distant targets (68%) relative to somewhat close (16%) or extremely close (16%) targets.

### Discussion

Consistent with our hypothesis, people view praise as more appropriate for actions that exceed a person’s duties compared with actions that are a person’s duties. That is, the judged praiseworthiness of an action is sensitive to the norms surrounding that action—specifically whether someone is obligated and expected to perform the action or whether performing the action would be outside of their typical obligations and expectations. Furthermore, the correlational analyses suggest that people see praise as less connected with the likelihood of doing the action and whether people should do the action, whereas blame is more connected with those considerations. Our results echo past research on how judgments of praise and blame are psychologically distinct (Wiltermuth et al., 2010). One implication from this study is regarding what general class of actions receive praise—that of supererogatory behaviors.

## Study 2

In Study 2, we provided participants with a short survey asking them to predict what would happen following praise or condemnation. We predicted that participants would hold the lay belief that praise serves to promote behavior, whereas condemnation would be perceived as serving to decrease the likelihood of a behavior.

### Method

#### Participants

We recruited and financially compensated 100 participants from Prolific. We did not collect any demographic information.

#### Procedure

Participants read: “Imagine that someone doesn’t know what is typically done in a situation: maybe they are in a new place and don’t know what people normally do here, or it is a new situation and they aren’t sure what is the ‘right’ thing to do.” Participants then answered two questions presented in random order, each starting with “They make a decision and do that action.” In one question, other people praise the individual for the action; in the other question, other people condemn the individual for the action. In both questions, participants were asked to predict whether the individual would repeat the behavior (*1 Definitely would not repeat what they did* to *7 Definitely would repeat what they did*).

### Results and Discussion

For both questions, we compared mean predictions against the midpoint of the scale (i.e., 4). On average, participants thought that the individual would be likely to repeat the behavior after receiving praise, *M* = 6.34, SD = 1.04, *t*(99) = 22.57, *p* < .001, *d* = 2.26. By contrast, participants that the individual would be unlikely to repeat the behavior after receiving condemnation, *M* = 1.93, SD = 1.93, *t*(99) = 17.25, *p* < .001, *d* = 1.73. Presentation order of whether participants answered the praise question or the condemnation question first did not moderate these effects, *F*(1,98) = 1.93, *p* = .17, *ƞ_p_*^2^ = .02. Together, these results provide an initial examination regarding the perceived consequences of praise and blame: as a general rule, praise is believed to increase a behavior, whereas condemnation decreases a behavior. We will contrast these results with those from Studies 4–6.

## Study 3

For the remaining studies, we transitioned to understanding people’s judgments regarding moral praise and its signaling value. Study 1 indicated people more strongly affirm the praiseworthiness of an action when it is supererogatory (i.e., being above and beyond the agent’s duties): People view praise as more appropriate for normatively exceptional actions than for duty-driven actions. With the remaining studies, we focus on the reverse set of inferences: If people are presented with a praised action, do they think that the action is more supererogatory?

In Study 3, we examined what information praise communicates to observers regarding norms. We employed a scenario taking place in a foreign country where the norms are relatively unknown. Without prior experience about how people tend to behave, would observers infer normative information when an action is praised? We predicted that in a novel cultural environment, moral praise for an act (vs. simple acknowledgment of the act and no acknowledgment of the act) would indicate that the behavior was relatively uncommon. We also investigated potential self–other differences in the effect of praise: would participants make different inferences between receiving praise versus observing praise for another? Given the long literature in actor–observer differences in judgment (Malle, 2006; Pronin et al., 2004), we examined whether norm inferences would generalize across different perspectives and different recipients of praise (i.e., the self vs. another person).

### Methods

#### Participants

We recruited 1,202 participants from Prolific. Given that we were primarily interested in the main effect of response type, we determined our sample size based on a power analysis to detect a main effect for a three-condition factor in an ANOVA of size *ƞ_p_*^2^ = 0.01 (a small effect, as recommended by G*Power) at power > .93 (Faul et al., 2009). Fifty-six people failed the attention check and were excluded, leaving a final sample of 1,146 (M_age_ = 32.03; 595 women, 526 men, 25 other), which still provides power > .90.

#### Procedure

Participants were randomly assigned to one of six conditions, based on a 2 (perspective: self, other) × 3 (response: praise, acknowledgment, control) between-subjects design. All participants were asked to imagine that they were visiting a foreign country and had only a basic understanding of local customs and practices. Participants then read that a couple of days into the trip, either they (in the *self* condition) or another person (in the *other* condition) help an elderly man carry his bags. A third party then comments on this behavior. In the *praise* condition, that person praises either the participant (*self* condition) or the other agent (*other* condition) for helping the elderly man. In the *acknowledgment* condition, the observer acknowledges that the participant (*self* condition) or the other person (*other* condition) helped the man but does not praise the action. In the *control* condition, the observer does not acknowledge or comment on what happened with the elderly man.

Participants then completed three measures (presented in random order). Participants estimated what percentage of people in this country would help an elderly person in need (from 0% to 100%). Participants completed two additional measures asking, in this country, how required is it that people help the elderly (from *1* = *Definitely not required* to *7* = *Definitely required*) and how expected is it that people help the elderly (from *1* = *Definitely not expected* to *7* = *Definitely expected*). Participants finally completed demographic items and an attention check (participants were instructed to write “purple” when asked what color the sky is).

### Results and Discussion

To assess the influence of praise on observer’s estimates of the descriptive and prescriptive norms, we conducted a 3 (response: praise, acknowledgment, control) × 2 (perspective: self, other) general linear model on each of our primary measures (See Table 3 for descriptive statistics). We also examined planned contrasts within each perspective condition, comparing the *praise* condition against each of the other two conditions, using Bonferroni corrections for two comparisons (see OSF page for full statistics). We predicted that the *praise* condition would be significantly different from the *acknowledgment* and *control* conditions on each of the measures; We were agnostic on whether the *acknowledgment* and *control* conditions would differ from each other. For zero-order correlations between variables, see Table 4.

**Table 3.** Study 3 Results.

		Praise	Acknowledgment	Control
Percentage estimate of people in this country who would help	Self	48.94 (23.48)	48.85 (23.98)	56.49^***^ (21.43)
	Other	50.89 (23.04)	60.02^***^ (21.35)	62.01^***^ (21.84)
How required is it that people help the elderly?	Self	2.99 (1.66)	3.35† (1.65)	3.81*** (1.61)
	Other	3.27 (1.75)	3.63† (1.73)	3.74^**^ (1.75)
How expected is it that people help the elderly?	Self	3.73 (1.65)	4.08† (1.57)	4.56*** (1.58)
	Other	4.09 (1.69)	4.65*** (1.59)	4.95*** (1.50)

*Note*. Means and standard deviations for each condition for the three primary measures. Superscripts summarize planned contrasts, comparing the *praise* condition against the *acknowledgment* and *control* conditions and using Bonferroni corrections for two comparisons. †: *p* < .10, *: *p* < .05, **: *p* < .01, ***: *p* < .001.

**Table 4.** Study 3 Correlations.

Variable	1	2	3
1. Percentage estimate of people in this country who would help	—		
2. How required is it that people help the elderly	.51	—	
3. How expected is it that people help the elderly?	.69	.63	—

*Note*. Zero-order correlation table showing the relations between all variables of interest in Study 3. All correlations significant at *p* < .001.

For estimates of the percentage of people in the country who would help, we found a main effect of perspective, *F*(1,1140) = 21.76, *p* < .001, *ƞ_p_*^2^ = .02, and the hypothesized main effect of response, *F*(2,1140) = 16.22, *p* < .001, *ƞ_p_*^2^ = .03. These main effects were qualified by an unanticipated significant interaction, *F*(2,1140) = 4.10, *p* = .02, *ƞ_p_*^2^ = .01. When imagining themselves as the moral agent, compared to no mention of the action by the bystander in the *control* condition, participants gave lower estimates for how many people in the country who would help after receiving either *praise, t*(569) = 3.19, *p* = .001, 95% CI [2.90, 12.20], or *acknowledgment, t*(569) = 3.24, *p* = .001, 95% CI [3.01, 12.28]. When imagining another person as the moral agent, participants gave lower estimates when the bystander praised the action compared to when the bystander simply acknowledged the action, *t*(571) = 4.05, *p* < .001, 95% CI [4.70, 13.56], or did not comment on it at all, *t*(571) = 4.90, *p* < .001, 95% CI [6.66, 15.57]. We did not predict this interaction a priori, and post-hoc power analyses suggest that our achieved power for this interaction was only moderate (power = .75, calculated using the “Superpower” package for R; Lakens & Caldwell, 2022). Given these concerns, we caution against overinterpreting. As speculation, this finding may suggest that people treat even acknowledgment of their own moral actions as praise, or at least information that the action is less common. However, when considering another person’s moral action, the action must be more explicitly praised before providing information about community norms. As expected for both perspectives, we found that observers interpreted praise for an action (vs. no mention of the action) to indicate that fewer people in the society performed that action.

For judgments of whether helping is required in the country, we found a significant main effect of response, *F*(2, 1138) = 13.61, *p* < .001, *ƞ_p_*^2^ = .02. There was no significant effect of perspective, *F*(1,1138) = 2.66, *p* = .10, *ƞ_p_*^2^ = .002, and no significant interaction, *F*(2, 1138) = 1.30, *p* = .27, *ƞ_p_*^2^ = .002. Supporting our hypothesis that praise provides normative information, for both the *self* and *other* conditions, praise for a moral action (vs. *acknowledgment* and *control*) was taken to indicate that the action was less required of people in the country.

For judgments of whether helping is expected in the country, we found a significant main effect of perspective: *F*(1,1138) = 21.49, *p* < .001, *ƞ_p_*^2^ = .02, whereby participants judged that the action was less required in the *self* condition than in the *other* condition. This makes sense, given that participants in the *other* condition may have interpreted the moral agent as a local, and therefore falling in the category of people “in this country.” Crucially for our hypotheses, we found the predicted main effect of response, *F*(1, 1138) = 26.48, *p* < .001, *ƞ_p_*^2^ = .04: for both *self* and *other* conditions, praise for an action, compared with simple acknowledgment or no mention, was interpreted to mean that the action was at least marginally less expected of people in the country. There was no significant interaction, *F*(1, 1138) = 0.46, *p* = .63, *ƞ_p_*^2^ = .001.

Our results extend Study 1, providing evidence that people treat moral praise as signaling behavioral norms. When a person does not know what the particular norms are for a situation, people infer from moral praise that an action is relatively uncommon and is less required or expected of people compared with no mention of the action and with simple acknowledgment of the action (although to a somewhat reduced degree).

## Study 4

In Study 4, we examined people’s beliefs regarding moral praise as a learning mechanism. In Study 2, we found that people hold the general lay belief that praise will increase the likelihood of a behavior. However, in more detailed contexts where they may be explicit reasons to behave less prosocially (e.g., self-interest), praise may ironically have an opposite effect. We compared the inferences people make for praise and for blame to determine whether these moral judgments are seen as having differential effects in shaping future behavior.

From Studies 1 and 3, people infer praised behaviors are less required and expected of the agent. Observers may predict that the agent is less likely to repeat the praised behavior, or at least not perform an action of greater prosociality (e.g., having been praised for donating $4, the agent does not then donate $5). The agent could simply do what they did again to maintain their positive evaluation. Praise may thus be seen as unlikely to change an agent’s behavior and potentially even lead to reductions in prosocial behavior. In contrast, blame indicates that an action was *not desired* and observers may predict that the agent *will* change their behavior because of the blame. Even young children recognize that people who are punished are less likely to transgress than those who are not punished (Bregant et al., 2016).

In Study 4, we examine the predictions observers make of an agent receiving either praise or blame (compared with a control neutral statement) for a donation behavior in an experimental economic game similar to the dictator game (e.g., Bardsley, 2008; Bolton et al., 1998). Across conditions, participants were placed in a third-party role and were presented with the same behavior where an agent is endowed with money (e.g., $10) and then distributes a slight majority of money to themselves (e.g., $6) and the remainder (e.g., $4) to another person. The agent then receives from a third-party observer praise, condemnation, or no evaluation. Participants then estimated the agent’s future behavior and the norms of the situation. As the primary test of our hypothesis, we predicted that praise, relative to a neutral statement and to blame, would reduce predictions of future behavior and assessments of the norms. As a secondary hypothesis, we also examined whether blame, relative to a neutral statement, would increase predictions of future behavior and assessments of the norms. We also considered participants’ own moral judgments of the situation and what they believed someone should do in the situation, although we did not have strong predictions in whether praise or blame (relative to a neutral statement) would change such judgments in a one-off scenario.

### Methods

#### Participants

We recruited 506 participants from Prolific and provided monetary compensation. We based this sample size to detect an effect size of *ƞ_p_*^2^ = .03 (the mean effect size for the *response* condition in Study 3) with power = .95. We excluded 65 participants for failing at least one of our comprehension checks, and one participant for not answering any of the dependent measures. Our final sample included 440 participants (M_age_ = 32.29; 236 women, 194 men, 10 other), which still provides power > .91.

#### Procedure

After providing consent, participants are introduced to an experimental economic game with three players. Each player is assigned a different anonymous role in the game: the Sender, the Receiver, and the Observer. The Sender receives $10 and then decides how to divide that money between themselves and the Receiver. The Receiver simply receives money given by the Sender. The Observer learns about the decision made by the Sender and can provide text-based feedback to them. After reading the rules, participants were asked three comprehension check questions: (1) “How much money is given to the Sender to start with?” (2) “Can the Receiver make any decisions in the game?” and (3) “What does the Observer do?” Participants had to correctly answer all three questions to be included in our analyses; participants who incorrectly answered any of these questions were automatically sent to the end of the study.

Participants were then randomly assigned to one of three between-subjects conditions. In all conditions, participants are external to the game; they learned that in the first round of the game, the Sender decided to give $4 to the Receiver and keep $6 for themselves, and then the Observer sent a message to the Sender. In the *praise* condition, the Observer expressed approval of the action (“That was really nice of you. If I was the Receiver, I’d be pretty happy.”). In the *blame* condition, the Observer expressed condemnation of the action (“That wasn’t very cool of you. If I was the Receiver, I’d be pretty mad.”). In the *control* condition, the Observer did not express any evaluation of the action (“First round finished.”).

Participants then completed four questions presented in randomized order. All questions were answered using a slider scale from $0 to $10 in $0.10 intervals. To measure participants’ estimates of behavioral change, we asked participants to predict how much money the same Sender would give in a second round of the game, with another $10 but new people as the Receiver and the Observer. To measure participants’ estimates of the descriptive norm, we asked participants to estimate how much money they think people in the position of the Sender generally give to the Receiver (i.e., what is the average amount of money given). To measure participants’ estimates of the prescriptive norms in the situation, we asked participants to indicate how much money they thought Receivers expect to get from Senders. Finally, we asked participants to indicate their own personal beliefs about the situation, asking them how much money they thought Senders should give to Receivers. After answering these four questions, participants completed a short demographics survey.

### Results and Discussion

Per our preregistration, we conducted an omnibus test to detect overall differences between conditions (see Table 5). For zero-order correlations between variables, see Table 6.

**Table 5.** Study 4 Results.

	Praise	Blame	Control	*F*(2, 437)	*p*	*ƞ_p_* ^2^
Second round estimation	4.00 (1.57)	4.49 (1.44)	3.97 (1.26)	6.26	.002	.03
Average amount sent by Senders	3.40 (1.66)	3.93 (1.35)	3.83 (1.36)	5.57	.004	.02
Amount that Receivers expect	3.58 (1.91)	4.63 (1.54)	4.33 (1.85)	13.75	< .001	.06
Amount that Senders should give	4.61 (1.28)	4.87 (1.18)	4.68 (1.39)	1.49	.23	.007

*Note*. Table displays means and SDs for each condition for the four primary measures, along with omnibus inferential statistics.

**Table 6.** Study 4 Correlations.

Variable	1	2	3	4
1. Second round estimation	—			
2. Average amount sent by Senders	.26	—		
3. Amount that Receivers expect	.24	.26	—	
4. Amount that Senders should give	.31	.18	.24	—

*Note*. Zero-order correlation table showing the relations between all variables of interest in Study 4. All correlations significant at *p* < .001.

Consistent with our prediction that the nature of the evaluative feedback would influence judgments of future behavior, we found a significant effect of condition on predictions of donation amount in a second round, *p* = .002. We next examined planned contrasts comparing different conditions against each other. As our main hypothesis, we examined whether the *praise* condition was significantly different from the *blame* and *control* conditions in estimates of second-round behavior. Unexpectedly, we found a nonsignificant contrast between participants who say a $4.00 donation be praised versus participants who saw the same behavior receive no judgment or receive blame, *t*(437) = 1.64, *p* = .10, 95% CI [−1.04, 0.10]. We next examined the secondary hypothesis of whether the *blame* condition would produce higher estimations of second-round behavior compared with the *control* condition. As expected, blame for a behavior did significantly increase judgments of future behavior compared with a neutral statement, *t*(437) = 3.13, *p* = .002, 95% CI [0.19, 0.85]. Given an initial behavior that was below an even distribution of the money (i.e., $5), participants anticipated that third-party blame and condemnation would prompt others to be *more* generous in the future.

We next examined whether, as we hypothesized, that praise (vs. the *blame* and *control* conditions) would influence judgments of the descriptive norm. As expected, we found a significant omnibus effect of condition on estimates of the average amount of money given by Senders, *p* = .004. Supporting our hypothesis that praise would reduce estimates of the descriptive norm, when the Sender was given third-party praise by the Observer for a $4.00 donation (compared with the *blame* and *control* conditions), participants estimated that Senders on average give relatively less money, *t*(437) = 3.27, *p* = .001, 95% CI [−1.55, −0.39]. Consistent with our previous studies, people interpret praise for an action as indicating that the action is relatively more prosocial than what people do on average (i.e., compared with the descriptive norm). Interestingly, participants in the *blame* and *control* conditions were not significantly different from each other, *t*(437) = 0.63, *p* = .53, 95% CI [−0.23, 0.44]. In addition, the means for the *blame* and the *control* conditions were not significantly different from the Sender’s original behavior of sending $4.00, *p*s > .13. Together, this pattern of results indicates that, when an action receives praise, the implied expectation is that people are on average less generous. When an action receives blame, the implied expectation is *not* that people are on average more generous: instead, participants estimated no significant difference between the statistical norm and the agent’s behavior.

We expected a similar pattern to emerge for judgments of the prescriptive norm and judgments of how much Receivers expect to receive. We found a significant difference between conditions on estimates on the amount of money that Receivers generally expect to receive, *p* < .001. Consistent with our hypothesis, when the $4.00 donation was praised (compared with the *blame* and *control* conditions), participants judged that Receivers expected relatively less money, *t*(437) = 5.03, *p* < .001, 95% CI [−2.51, −1.10]. One possible interpretation of this finding is that participants interpreted the Observer’s comments as a reaction for how that person would have felt in the position of the Receiver. Thus, participants would have taken the Observer and their reaction as a potential substitute for how Receivers in general would think and feel. The *blame* and *control* conditions were not significantly different from each other, *t*(437) = 1.46, *p* = .15, 95% CI [−0.10, 0.71].

For participants’ personal beliefs of how much Senders should give, we found no overall difference between conditions and no differences from our planned contrasts, *p*s > .23.

Finally, we tested our hypothesized mechanism—that praise would lead to lower estimates of the perceived norms, and that perceived norms would in turn influence behavioral intentions. We created two dummy-coded variables comparing *praise* (coded as 1) against the *blame* and *control* conditions (both coded as 0). To form a composite index of perceived norms, we averaged together participants’ estimates of the descriptive norm (i.e., the average amount sent by Senders) and the prescriptive norm (i.e., the amount expected by Receivers). We conducted two mediation models (comparing *praise* separately against *blame* and *control*) using PROCESS Macro 4 (Hayes, 2012) with 10,000 bootstrapped samples, assigning the dummy-coded variables as the independent variable, second-round estimation as the dependent variable, and the estimated average amount sent by Senders as the mediator. We found significant mediation when comparing *praise* against both the *blame* condition, *b* = −0.31, SE = .09, 95% CI [−0.51, −0.15], and the *control* condition, *b* = −0.20, SE = .07, 95% CI [−0.34, −0.08]. This suggests that praise lowers estimates of the perceived norms compared with blame and neutral statements, which can contribute to reduced predictions of future behavior.

## Study 5

With Study 5 we aimed to replicate and extend the results of our previous studies. As in Study 3, we asked participants to imagine themselves in a foreign country where they are unfamiliar with the local norms. Participants were told that they performed a voluntary, prosocial action (e.g., tipping someone for their service) and then that behavior received either praise, blame and condemnation, or no evaluation from a third party. As in previous studies, we predicted that praise (relative to blame and a neutral control) would lead to reduced perceptions of the norms (i.e., the behavior being less common, required, and expected of people). As a secondary hypothesis, we examined whether blame (relative to neutral control) would lead to heighted perceptions of the norms.

We also examined two new judgments, both relating more specifically to moral judgment and moral behavior. First, we asked participants to evaluate how morally acceptable it would be to perform a similar action of reduced magnitude in the future. If, as predicted, praise makes an action seem less normative, then observers may make the subsequent inference that it would be morally acceptable to be *less* moral in the future (Lindström et al., 2018; Ngo et al., 2015). Alternatively, blame and condemnation for an action may have the opposite effect: as a sanction on that specific behavior, being even less moral would invite an equal, if not greater, amount of blame. We predicted that praise for the action (relative to a neutral statement and to blame) would make it seem more morally acceptable to behave less moral, whereas blame for the action would make it seem less morally acceptable to be less moral (relative to a neutral statement).

Second, we asked participants to predict whether they would behave as prosocially in a similar situation in the future. Like judgments of moral acceptability, we predicted that if the praised behavior is seen as less normative, then participants may feel licensed to behave less morally in the future. As outlined above, praise for an action may unwittingly promote less moral behavior (relative to a neutral statement and to blame) because actions of that magnitude are interpreted as being less required and expected of people. When predicting their future behavior, observers may then infer that they could be less prosocial and not suffer any social reprimands.

### Methods

#### Participants

We recruited 597 participants from Amazon’s Mechanical Turk using CloudResearch’s prime panels (Chandler et al., 2019) and provided them with monetary compensation. We based our sample size on the observed effect size of the *response* condition from Study 3 at power > .95. We excluded 17 participants for failing our attention check, leaving a final sample of 580 (*M_age_* = 39.23; 335 men, 237 women, three greygender/nonbinary/queer, five blank), which still provides power > .95.

#### Procedure

Participants were randomly assigned to one of three between-subjects conditions. In all conditions, participants read a vignette that told them that they were visiting a friend in a foreign country that they have never visited before (for full text, see our OSF page). The friend tells the participant to take a taxi to the friend’s house, for which the friend will cover the cost. The vignette says that the participant is new to this country and unsure of whether and how much to tip the driver, but decides to tip the taxi driver 15% of the total cost. After meeting with the friend, the friend pays the participant back for the regular taxi fare, but the participant is said to cover the tip themselves. The vignette then says that the participant has dinner with the friend and the friend’s wife. While in another room, the participant overhears the friend telling the wife about the taxi ride and covering the fare. The friend mentions to the wife how much the participant tipped to the taxi driver. In the *praise* condition, the wife praises the participant’s tipping behavior, saying that it was “nice” and “I bet that driver was quite happy about that.” In the *blame* condition, the wife condemns the participants’ tipping behavior, saying that it was “pretty rude” and “I bet that driver was upset about that.” In the *control* condition, the wife simply expresses that she is glad that it worked out and that the participant arrived okay.

Participants then responded to five questions. Participants estimated what percentage of people in this country they thought tip their taxi drivers at least 15% (from 0% to 100%). Participants completed two additional measures asking, in this country, how much people feel required to tip their taxi drivers and how much people expect others to tip their taxi drivers (both from *1* = *Not at all* to *7* = *Definitely*). In one question, participants were asked to imagine themselves taking the taxi again to return to the airport and to estimate how morally acceptable it would be for them to tip less than 15% of the fare to the driver this time (from *1* = *Not acceptable to give less than 15%* to *7* = *Very acceptable to give less than 15%*). Finally, participants were asked to imagine themselves visiting this country again and if they were to take the taxi again, to estimate how much they would tip the driver this time (from *1* = *Much less than before* to *7* = *Much more than before*). Participants finally completed demographic items and the same attention check measure as Study 3.

### Results and Discussion

Per our preregistration, we conducted an omnibus test to detect overall differences between conditions (Table 7). For zero-order correlations between our measures, please see Table 8. We followed a similar analytic plan as Study 4.

**Table 7.** Study 5 Results.

	Praise	Blame	Control	*F*(2, 576)	*p*	*ƞ_p_* ^2^
Percent in country who tip at least 15%	26.42 (20.73)	45.77 (32.82)	50.94 (23.67)	46.84	< .001	.14
How required do people feel to tip	3.04 (1.73)	5.05 (2.11)	4.42 (1.55)	62.06	< .001	.18
Do people expect others to tip	2.95 (1.68)	5.27 (2.06)	4.53 (1.64)	83.50	< .001	.22
Morally acceptable to give less than 15%	5.28 (1.62)	3.10 (2.16)	3.81 (1.72)	69.91	< .001	.20
Future tipping behavior	3.48 (1.24)	4.77 (1.69)	3.95 (0.93)	46.25	< .001	.14

*Note*. Table displays means and SDs for each condition for the five primary measures, along with omnibus inferential statistics. The first measure ranged from 0% to 100%; all other items were on a 1–7 scale.

**Table 8.** Study 5 Correlations.

Variable	1	2	3	4	5
1. Percent in country who tip at least 15%	—				
2. How required do people feel to tip	.61	—			
3. How much do people expect others to tip	.64	.86	—		
4. Morally acceptable to give less than 15%	–.45	–.55	–.54	—	
5. Future tipping behavior	.44	.62	.62	–.56	—

*Note*. Zero-order correlation table showing the relations between all variables of interest in Study 5. All correlations significant at *p* < .001.

Consistent with our predictions, we found a significant omnibus effect of condition on estimates of the percentage of people in the country who tip their taxi drivers at least 15%, *p* < .001. Supporting our prediction that praise would lead to reduced estimates for the statistical norm of a behavior, participants in the *praise* condition had the lowest mean estimate compared to the other conditions, *t*(576) = 9.46, *p* < .001, 95% CI [−52.98, −34.77]. We also predicted that blame may lead to estimates that the behavior was more common than a neutral statement, although we actually found a marginally significant difference in the other direction such that participants in the *blame* condition made lower estimates of the percentage of people in the country who tip compared with the *control* condition, *t*(576) = 1.94, *p* = .052, 95% CI [−10.40, 0.05].

For judgments of what people feel required to do and what people expect others to do, we found a significant effect of condition on both items, *p*s < .001. As hypothesized, participants in the *praise* condition (relative to the *blame* and the *control* conditions) gave the lowest mean judgments for what is required, *t*(577) = 10.62, *p* < .001, 95% CI [−4.02, −2.76], and what is expected, *t*(577) = 12.30, *p* < .001, 95% CI [−4.53, −3.29]. Consistent with our predictions and our previous studies, praise for an action communicates that the action is generally seen as less required and less expected of people. Consistent with our prediction, participants in the *blame* condition, versus the *control* condition, made significantly higher estimates of whether people feel required to do the action, *t*(577) = 3.44, *p* < .001, 95% CI [0.27, 0.99], and whether they expect others to do the action, *t*(577) = 4.04, *p* < .001, 95% CI [0.38, 1.10]. In contrast to praise, blame and condemnation for an action makes the action seem more required and expected of people.

For judging whether it was morally acceptable to give less than 15% tip on a future taxi trip, we found that there was a main effect of condition, *p* < .001. Supporting our hypothesis that praise may influence how morally acceptable less generous behaviors are, we found that participants in the *praise* condition judged that tipping less than 15% on a future taxi trip was more morally acceptable than participants in the other two conditions, *t*(577) = 11.173.44, *p* < .001, 95% CI [3.00, 4.28]. Consistent with our prediction that blame would operate in the opposite direction as praise, we also found that participants in the *blame* condition judged that tipping less than 15% would be less morally acceptable than participants in the *control* condition, *t*(577) = 3.80, *p* < .001, 95% CI [−1.08, −0.34].

As expected, we found a significant main effect of condition on predictions of future behavior, *p* < .001. We predicted that praise for the action would lead to lower estimates of future behavior compared to blame and to the control statement. Supporting this hypothesis, participants in the *praise* condition (vs. the other conditions) predicted that they would reduce the magnitude of their action, acting in a less prosocial manner than the previously praised action, *t*(577) = 7.56, *p* < .001, 95% CI [−2.22, −1.31]. In this way, praise may potentially lead people to behave less morally than they had before. In contrast, participants in the *blame* condition predicted that they would increase the magnitude of the action relative to the *control* condition, acting in a more prosocial manner than the previously condemned action, *t*(577) = 6.07, *p* < .001, 95% CI [0.55, 1.08]. Both praise and blame can modify future behavior, but praise may unwittingly *reduce* the magnitude of future moral actions similar to the initially praised action.

As in Study 4, we tested for statistical mediation of the norm mechanism. We z-scored and averaged together the three norm judgments (percent who tip, required to tip, expected to tip; α = .88). Otherwise, we followed the same procedure as in Study 4, conducting two mediation models comparing the *praise* condition separately against the *blame* and the *control* conditions. We found significant mediation when comparing *praise* against both the *blame* condition, *b* = −1.05, SE = .12, 95% CI [−1.30, −0.83], and the *control* condition, *b* = −0.57, SE = .08, 95% CI [−0.74, −0.41]. Supporting our overall theory, these mediation results suggest that judgments of the perceived norms resulting from praise have a significant dampening effect on intentions to behave prosocially in the future.

## Study 6

Study 6 was a close replication of Study 5, with several modifications. We used a slightly different behavior—tipping at a restaurant—to extend the generalizability of the effect. In addition, we included manipulation checks of perceived praise and blame. We hypothesized the same pattern of results as in Study 5, such that praise (relative to a neutral control and to blame) would lead to reduced estimates of the norms and predictions of future behavior. Less critical to our central theory, we also considered whether blame (relative to a neutral control) would lead to heightened estimates of the norms and predictions of future behavior.

### Method

#### Participants

We recruited 300 participants through Prolific (180 women, 113 men, one agender, one gender queer, four nonbinary, one other; *M_age_* = 40.01, SD_age_ = 12.51). We based this sample size on the observed effect sizes of Study 5 with power > .80. Four participants failed the attention check—to maximize power, we retain the full sample in the analyses as the conclusions do not change when we excluded these participants.

#### Procedure

Study 6 was identical to Study 5, with several exceptions (see OSF page for full text). We modified the vignette to read that instead of taking a taxi, the participant instead ate at a restaurant and tipped the servers 15% of the bill. As in Study 5, participants were randomly assigned to a *praise* condition in which the friend’s wife praises their tipping behavior (“That was really nice of your friend”), a *blame* condition in which the friend’s wife condemns their tipping behavior (“That was pretty rude of your friend”), or a *control* condition in which there is no direct comment on the tipping behavior. After the vignette, participants completed three primary measures: the estimated percentage of people who tip at least 15% at restaurants (from 0% to 100%); how much people expect others to tip restaurant servers (from *1* = *Not at all* to *7* = *Definitely*); and if they were to eat at a restaurant in this country again, how much they would tip the server this time (from *1* = *Much less than before* to *7* = *Much more than before*). Participants also completed two manipulation check items: how much it sounded like what the friend’s wife said was (a) praising them or (b) condemning them for what they did (both from *1 Not at all* to *7 A great deal*). Participants then reported demographics and completed the same attention check item as in Study 3.

### Results and Discussion

As in Study 5, we conducted a series of omnibus ANOVAs (see Table 9; for zero-order correlations, see Table 10). Per our manipulation checks, we successfully manipulated praise in the *praise* condition and condemnation in the *condemnation* condition, *p*s < .001. Replicating Study 5, we again found that praise—relative to the *blame* and *control* conditions—led to reduced estimates of the percentage of people performing a behavior, *t*(296) = 5.20, *p* < .001, 95% CI [−48.91, −22.07]; led to lower estimates of expectations, *t*(296) = 6.50, *p* < .001, 95% CI [−3.87, −2.07]; and reduced the predicted degree of prosocial behavior in similar future situations, *t*(296) = 2.90, *p* = .004, 95% CI [−1.88, −0.36]. When comparing the *blame* and the *control* conditions, we found no significant difference for how expected the behavior was, *t*(296) = 0.20, *p* = .98, 95% CI [−0.51, 0.52], and judgments of future behavior, *t*(296) = 0.72, *p* = .47, 95% CI [−0.28, 060]. In addition, judgments of the frequency of the behavior were actually in the opposite direction as we predicted, such that participants in the *blame* condition made lower estimates of the percentage of people who perform the behavior compared to the *control* condition, *t*(296) = 2.35, *p* = .02, 95% CI [−16.84, −1.47].

**Table 9.** Study 6 Results.

	Praise	Blame	Control	*F*(2, 297)	*p*	*ƞ_p_* ^2^
Praise manipulation check	5.57 (1.17)	1.28 (0.82)	3.04 (1.70)	278.23	< .001	.65
Condemnation manipulation check	1.60 (1.25)	6.14 (1.16)	1.90 (1.29)	420.95	< .001	.74
Percent in country who tip at least 15%	27.32 (21.12)	40.35 (34.59)	59.51 (25.43)	16.25	< .001	.10
Do people expect others to tip	3.04 (1.32)	4.52 (2.40)	4.51 (1.68)	21.03	< .001	.05
Future tipping behavior	3.48 (1.23)	4.12 (2.17)	3.96 (1.08)	4.42	.01	.03

*Note*. Table displays means and SDs for each condition for the two manipulation checks and the three primary measures, along with omnibus inferential statistics. The third measure went from 0% to 100%; all other items were on a 1–7 scale.

**Table 10.** Study 6 Correlations.

Variable	1	2	3	4	5
1. Praise manipulation check	—				
2. Condemnation manipulation check	–.63***	—			
3. Percent in country who tip at least 15%	–.12*	.01	—		
4. Do people expect others to tip	–.16**	.15**	.73***	—	
5. Future tipping behavior	–.02	.07	.62***	.74***	—

*Note*. Zero-order correlation table showing the relations between all variables of interest in Study 6. * *p* < .05, ** *p* < .01, *** *p* < .001.

As in Studies 4 and 5, we tested the mediating effect of perceived norms on future behavior, first z-scoring the percent estimate and expectation items and then computing the average of these z-scores as our measure of perceived norms. We conduct two sets of mediation models, comparing the *praise* condition against either the *blame* condition or the *control* condition. We found significant mediation when comparing *praise* against both the *blame* condition, *b* = −0.90, SE = .19, 95% CI [−1.25, −0.52], and the *control* condition, *b* = −0.67, SE = .12, 95% CI [−0.92, −0.46]. Together with Studies 4 and 5, these results highlight how praise can *lower* estimates of perceived norms, which can in turn *lower* intentions to behave prosocially in the future.

## General Discussion

The present studies aimed to address two open questions regarding the nature of moral praise. First, we examined whether one potential dimension by which an action becomes praiseworthy is by exceeding the agent’s duties and responsibilities (as predicted by recent theorization; Anderson et al., 2018, 2020). In Study 1, we found that participants judged actions that exceed a person’s duties, compared with a person’s duties, as being more praiseworthy. This suggests that certain actions are judged as praiseworthy because of their supererogatory nature, with implications for how common and expected praised acts are seen to be.

Second, we examined the inferences people draw from seeing an act receive moral praise. As a baseline, people hold the belief that praise reinforces the praised behavior (Study 2). However, in more specific scenarios, we found that people infer information about the norms surrounding the action (Studies 3–6). Building upon its theorized functions (Anderson et al., 2020; Schein et al., 2020), our work highlights that praise may signal norms of whether certain prosocial actions are expected and required of people. We consistently found that praise for an action, compared with acknowledgment and blame, led observers to infer that the action was less common, expected, and required of people. Finally, we found that praise for an action may potentially influence moral judgment and moral behavior (Studies 5–6), leading participants to judge that less prosocial actions are more morally acceptable and to predict that they would behave less morally in the future.

It is worth noting the somewhat inconsistent results between Study 4 and Studies 5–6 in comparing the *praise* condition and the *control* conditions; Study 4 did not find a significant difference, but there was one in Studies 5–6. One possibility is that the stimuli in Studies 5–6 (and Study 3 for that matter) were framed as being about a new cultural context, whereas in Study 4 the situation (while potentially novel) is still within the participants’ cultural framework. Future work can explore more directly how knowledge of cultural norms may moderate our effect.

### Implications

We believe our findings have important implications for how and what people learn from social and moral judgments like praise. Specifically, our work demonstrates that people infer that praise does not simply provide information about the value or desirability of an action (i.e., whether some action is *good*). Instead, praise provides information about the surrounding normative context for the action: how statistically common that action is and whether people are required or expected to do that action. Praise may thus communicate what the baseline or reference behavior is, and such reference points can have important implications for how people construe their social environment (Chestnut & Markman, 2018). By seeing attention drawn to certain behaviors through praise, observers may learn about what is and is not expected and required. Such information may be especially important for children’s development: when parents praise a moral action of their child, the children could infer from the praise that the action is unexpected and potentially unnecessary to perform. Past work has similarly found that rewarding children’s prosocial behavior can lead to reductions in such behavior as children ascribe motivation to the reward itself (Fabes et al., 1989). Understanding how best to apply praise (and other rewards) can thus be important for moral education in children.

Likewise, praise may unwittingly *reduce* the extremity of moral behaviors: by making a behavior seem less required and expected of people, praise may license agents to behave less prosocially and more selfishly. This ironic effect runs counter to lay beliefs about what happens following praise (Study 2): As a baseline, people expect praise to reinforce a behavior. However, in more concrete vignettes, where the tension between behaving prosocially and selfishly is made more apparent (e.g., giving money to another vs. keeping it for the self, Studies 5–6), praise lowers estimations of the norms and may thus enable people to behave selfishly instead of prosocially.

While our studies have focused on praise for moral actions, we believe that our findings can speak to praise more generally. There is a large literature on praise and feedback for competence-based achievements (e.g., work performance, academics, athletic and artistic skills), especially in the context of motivation and learning (e.g., Brett & Atwater, 2001; Henderlong & Lepper, 2002; Mueller & Dweck, 1998). While research on praise for moral behaviors and for achievement behaviors frequently operates in isolation, we believe that our findings would apply equally well for the latter behaviors. For example, praising someone for their artistic accomplishment likely communicates similar information about norms and expectations regarding that behavior: that such achievements are relatively uncommon and not necessarily expected of the agent.

### Future Directions

Our studies provide an initial examination of the interplay between moral norms and moral praise. Given that we focused on the informative nature of praise, it may be that there are differences in the source of praise that make it more informative. For instance, group leaders can have an especially powerful impact on group norms (Crandall et al., 2018; Lemoine et al., 2019; Munger, 2017; Padilla et al., 2007). Praise from a group leader may provide a particularly clear signal of the group’s norms. In addition, work on feedback in achievement contexts (e.g., Bryan et al., 2014; Mueller & Dweck, 1998) has distinguished between feedback for the *act* (e.g., “this is good/bad) versus the *person* (e.g., “you are good/bad”). As our studies did not systematically test this distinction, future work could explore whether act-focused versus person-focused praise may have different effects on perceived norms.

Another open question is how praise relates to other socially oriented positive evaluations like gratitude (which may also at times reflect a moral evaluation). Offering praise (e.g., “you’re a good person”) and offering gratitude (e.g., “thank you for what you did”) may both reflect a similar sentiment and may therefore have similar communicative value. In a pilot study (N = 99) conducted on Prolific, we asked participants to describe what they would say if they were to praise a person for doing something morally good. We found that 37% of participants included mention of thanking that individual, suggesting that people frequently think of “praise” and “gratitude” in similar ways. Future work should more directly examine similarities and differences between expressions of praise, expressions of gratitude, and other positive social evaluations.

## Conclusion

Moral praise is a rich social judgment that both responds to and reflects normative considerations. It communicates expectations and ethical norms, and its role in reinforcing behavior may be less straightforward than previously thought.
